# Tumour Necrosis Factor-α Production and Immune Cell Activation in Tuberculous Meningitis

**DOI:** 10.1155/S0962935194000104

**Published:** 1994

**Authors:** C. M. Mastroianni, F. Paoletti, C. Valenti, A. P. Massetti, V. Vullo, S. Delia

**Affiliations:** Institute of Infectious Diseases University ‘La Sapienza’ Policlinico Umberto I Viale Regina Elena 331 Rome 00161 Italy

## Abstract

The local production of tumour necrosis factor-α (TNFα) was evaluated in the cerebrospinal fluid (CSF) from ten patients with tuberculous meningitis (TBM). The degree of intrathecal immune activation was also studied by assessing the CSF levels of β_2_-microglobulin (β_2_-M) and adenosine deaminase activity (ADA). Results indicate that elevated CSF concentrations of TNFα, β_2_-M and ADA were found in all TBM patients. Moreover, TNFα is produced and selectively concentrated for a long period of time, while β_2_-M and ADA values progressively decline during the course of TBM. Our findings suggest that in TBM patients, after an early activation of immune cells, there is an enhanced and continuous production of TNFα at the site of infection.


Mediators of Inflammation 3, 57–60 (1994)

### Tumour necrosis factor-α production and immune cell activation in tuberculous meningitis

C. M. Mastroianni,[CA] F. Paoletti, C. Valenti, A. P. Massetti, V. Vullo and S. Delia

Institute of Infectious Diseases, University 'La Sapienza', Policlinico Umberto I, Viale Regina Elena 331, 00161 Rome, Italy

[CA] Corresponding Author

**THE local production of tumour necrosis factor-α (TNFα) was evaluated in the cerebrospinal fluid (CSF) from ten patients with tuberculous meningitis (TBM). The degree of intrathecal immune activation was also studied by assessing the CSF levels of $\beta_2$-microglobulin ($\beta_2$-M) and adenosine deaminase activity (ADA). Results indicate that elevated CSF concentrations of TNFα, $\beta_2$-M and ADA were found in all TBM patients. Moreover, TNFα is produced and selectively concentrated for a long period of time, while $\beta_2$-M and ADA values progressively decline during the course of TBM. Our findings suggest that in TBM patients, after an early activation of immune cells, there is an enhanced and continuous production of TNFα at the site of infection.**

**Key words:** Adenosine deaminase, $\beta_2$-microglobulin, Cerebrospinal fluid, Immune activation, Tuberculous meningitis, Tumour necrosis factor-α

### Introduction

Tumour necrosis factor-α (TNFα) is a cytokine mainly released by cells of the monocyte-macrophage lineage in response to lipopolysaccharide (LPS) and other immune and inflammatory stimuli. It has been recognized as a primary mediator in the pathogenesis of infection, injury and inflammation and in the beneficial processes of host response.[1] In particular, TNFα is an important macrophage-activating factor for antibacterial resistance against infections caused by facultative intracellular organisms, such as mycobacteria.[2]

In experimental models it has been reported that TNFα enhances macrophage phagocitic capacity and mycobacterial killing by human macrophages.[3] In addition, recent lines of evidence indicated that TNFα is synthesized in large amounts in pulmonary tuberculosis and it is locally concentrated at the site of disease activity.[4,5] To date, little is known about the intrathecal production of TNFα during the course of tuberculous meningitis (TBM).[6] Therefore, it appears to be of interest to evaluate the local production and release of TNFα in the cerebrospinal fluid (CSF) of patients with TBM.

In the present study, TNFα concentrations were measured in CSF specimens obtained from hospital patients on admission and during the course of TBM. In order to determine the extent to which cell-mediated immunity is involved in the disease, the CSF levels of two markers of immune activation, such as $\beta_2$-microglobulin ($\beta_2$-M) and adenosine deaminase activity (ADA), were also measured.

### Materials and Methods

*Patients:* Ten patients with TBM (five males, five females) admitted to the Institute of Infectious Diseases of the University of Rome 'La Sapienza', were enrolled in this study. The mean age was 34.4 years (range, 12–55 years). TBM was diagnosed on the basis of findings in the CSF. In six cases CSF smears were positive by acid-fast staining and fluid cultures grew *Mycobacterium tuberculosis*; in the remaining patients, diagnosis was made by compatible cytological and biochemical findings, supported by clinical features and symptomatic improvement with antituberculous therapy. All patients were sero-negative for human immunodeficiency virus (HIV) and did not have other disturbances of immunity.

A total of 66 CSF samples was collected from TBM patients before starting treatment and subsequently at various intervals of time during the course of infection.

CSF specimens were also obtained from 15 patients with non-infectious neurological disorders, including cerebrovascular disease, hydrocephalus, encephalomyelitis and epilepsy.

CSF specimens, upon collection, were placed in a refrigerated centrifuge (4°C) and spun at 3 000 rpm for 10 min. Cell supernatants were stored at −80°C until use.

*Assay for TNFα:* TNFα concentrations in CSF were measured by a quantitative immunoenzymatic sandwich assay (Quantikine, R&D Systems, Minneapolis, MN, USA). Samples and standards were

© 1994 Rapid Communications of Oxford Ltd incubated in anti-TNFα monoclonal antibody-coated polystyrene microtiter wells. A horseradish peroxidase-conjugated anti-TNFα polyclonal antibody was then added and allowed to bind the TNFα captured by the first antibody, completing the sandwich. After washing, substrate solution was added to the wells. The reaction was stopped by addition of 2N $H_2SO_4$ and optical density (OD) at 492 nm was measured using an ELISA microreader. To estimate the amount of TNFα in the CSF samples we constructed a standard curve by using concentrations of TNFα ranging from 0 to 1 000 pg/ml. Unknown values of TNFα in the samples were determined by referring to the standard curve and expressed as pg/ml. The detection limit of the assay was 4.8 pg/ml.

*Assay for $\beta_2$-M:* $\beta_2$-M concentrations were assessed by the IMx system (Abbott Laboratories, North Chicago, IL, USA), according to the manufacturer's instructions. The IMx system is based on a rapid microparticle enzyme immunoassay (MEIA), in which submicron particles are coated with a mouse monoclonal antibody specific for $\beta_2$-M. The use of microparticles increases the assay kinetics and decreases the assay incubation. The sensitivity of the IMx $\beta_2$-M was calculated to be better than 5 $\mu$g/l. Normal $\beta_2$-M values were considered equal or less than 3 mg/l.

*ADA activity measurement:* ADA activity was measured in fresh CSF samples after centrifugation at 300 *g* for 10 min. ADA assay was performed at 37°C by using the colorimetric method of Giusti,[7] based on the determination of the amount of $NH_3$ released in the reaction mixture. The enzymatic activity was calculated by referring to a standard curve of ammonium sulphate in buffer and was expressed as IU/37°C/l. Normal ADA values were considered equal or less than 4 IU/l.

*Statistical analysis:* The Mann-Whitney *U*-test was used for statistical analysis.

### Results

CSF levels of TNFα, $\beta_2$-M and ADA were measured in ten patients with TBM and in 15 control subjects with non-infectious CNS disorders. Data are shown in Figure 1 and are expressed as mean ± SEM. TNF α concentrations in TBM patients (155 ± 21.62 pg/ml) were significantly higher than those found in controls (5.69 ± 0.77 pg/ml) ($p < 0.0001$). Similarly, TBM patients had elevated CSF values of $\beta_2$-M (8.69 ± 1.07 mg/l) and ADA (10.83 ± 1.61 IU/37°C/l) in comparison with those observed in the non-TBM cases (1.34 ± 0.12 mg/l and 1.02 ± 0.16 IU/37°C/l, respectively) ($p <$ 0.0001 for both).

The kinetics of TNFα and both $\beta_2$-M and ADA values during the course of TBM were illustrated respectively in Figures 2 and 3.

In all patients CSF levels of TNFα were low on admission, then progressively increased and remained detectable for a long period of time. Antibiotic treatment did not appear to influence the TNFα levels. Similarly, there was no correlation between the cytokine concentrations and the clinical course of the disease.

On the contrary, $\beta_2$-M and ADA values resulted to be elevated on admission. Later, a progressive decline was observed. Only two patients with culture-proven TBM showed a further increase in CSF $\beta_2$-M and ADA values, despite the administration of antituberculous treatment. The peak of these

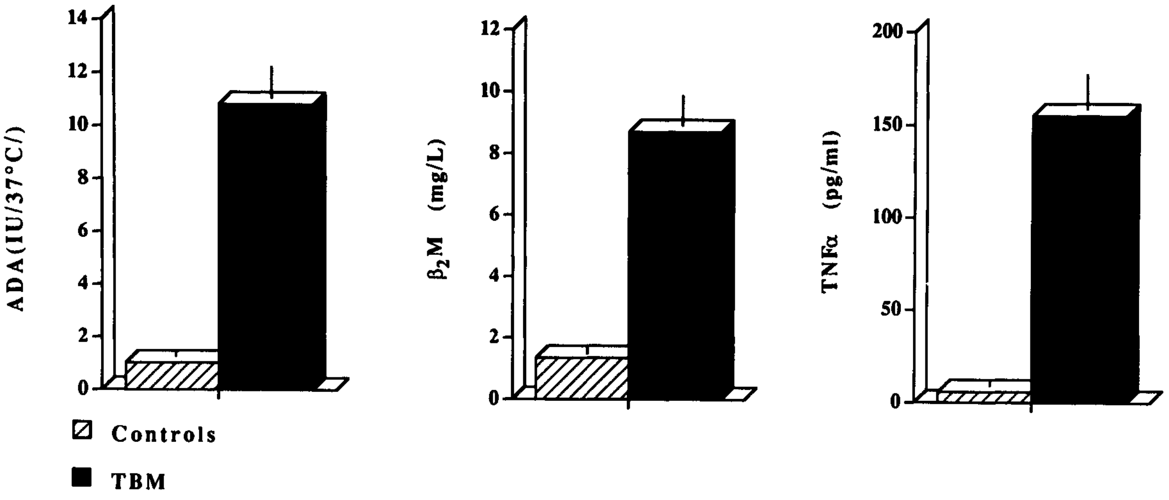

FIG. 1. Levels of $\beta_2$-M, ADA and TNFα in the CSF samples ($n = 66$) from TBM patients compared with those ($n = 15$) obtained in controls with non-infectious neurological diseases. Data are expressed as the mean ± SEM. Comparisons between groups are made by non-parametric test (Mann–Whitney *U*-test) and differences in CSF levels of $\beta_2$-M, ADA and TNFα were statistically significant ($p < 0.0001$ for all).

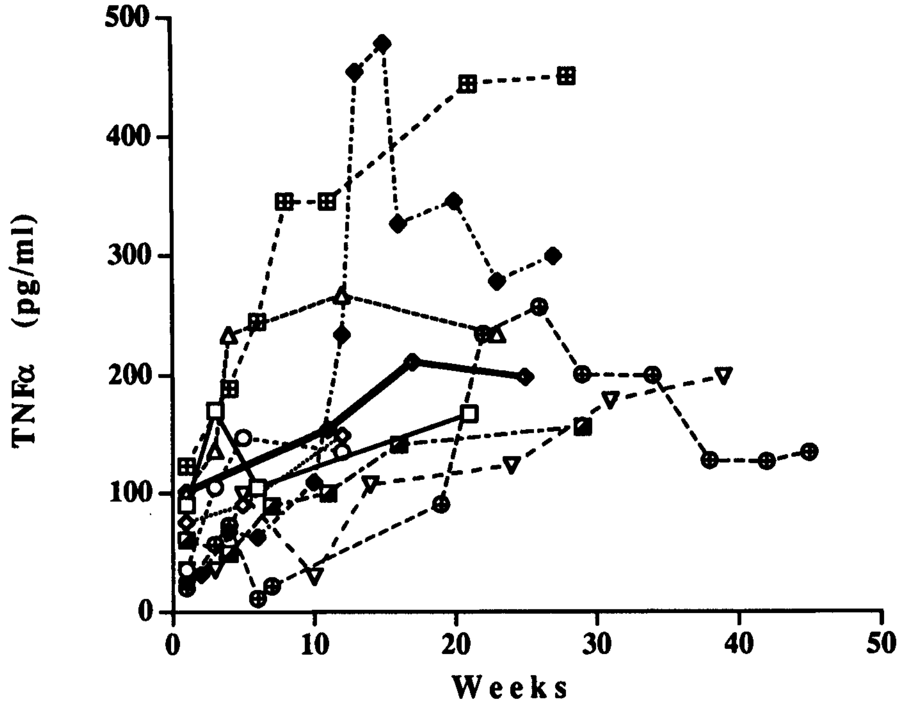

FIG. 2. Time course of TNFα levels in sequential CSF samples from the ten TBM patients.

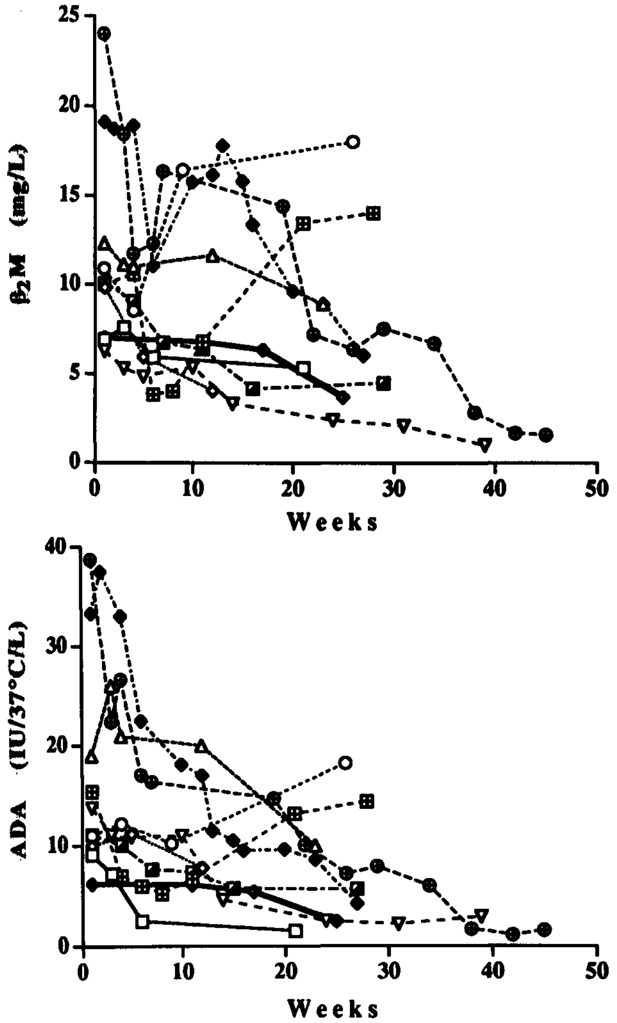

FIG. 3. Time course of $\beta_2$-M and ADA levels in sequential CSF samples from the ten TBM patients.

immune activation markers was associated with the development of complications (hydrocephalus, hemiplegia) and an unfavourable clinical course of meningitis.

### Discussion

Cell-mediated immunity plays an important role in the control of infections by *M. tuberculosis*. It is widely assumed that T-lymphocytes, through lymphokine-mediated macrophage activation, are the major immune cells involved both in the pathogenesis and protective mechanisms in human tuberculosis.[8–10]

In the case of TBM, it is conceivable that the involvement of cellular immune system mainly occurs in the central nervous system (CNS). In this respect, the dosage in the CSF of two markers of immune activation, such as $\beta_2$-M and ADA, represents a useful tool to investigate the degree to which cell-mediated immunity is stimulated in the CNS. $\beta_2$-M is a portion of the major histocompatibility complex class I antigen (MHC-1) and is expressed on the surface of lymphocytes and macrophages. With regard to the adenosine deaminase, the activity of this enzyme is increased in lymphoid cells, especially during T-cell proliferation and differentiation.

Previous investigations have already demonstrated a close correlation between elevated CSF ADA values and TBM,[11,12] while an increase in the CSF concentrations of $\beta_2$-M has been reported only in viral and pyogenic meningitis.[13] In the present study, both $\beta_2$-M and ADA levels were found to be elevated in the CSF of all TBM patients, thus indicating a marked stimulation of immune system within the CNS. The increased CSF levels of $\beta_2$-M were possibly a consequence of cell-mediated cytotoxicity directed against macrophages infected by *M. tuberculosis*.[14] The elevation of CSF ADA values was most likely related to the local immune response as the result of proliferating lymphocytes in response to mycobacterial antigen.

Intrathecal immune activation appears to be also associated with increased cytokine expression in the CNS. Indeed, our results showed that TBM patients had significantly higher CSF TNFα concentrations than those found in control subjects with non-infectious neurological diseases. The local production of TNFα is low in the initial phase of TBM, while the peak in the CSF concentration was obtained later. Moreover, the CSF levels of this cytokine did not decrease during the course of the disease, but they were elevated for a long time in all patients, irrespective of the antibiotic therapy and the clinical course of TBM. This pattern is quite different from that observed in the case of $\beta_2$-M and ADA, whose levels declined rapidly during the course of the disease. Only two patients who developed neurological complications showed a further increase in the CSF $\beta_2$-M and ADA values.

Taken together, our findings suggest that a great stimulation of immune T-cells primarily occurs in the acute phase of TBM and may account for the early increase in the CSF $\beta_2$-M and ADA values. On the contrary, the enhancement of cytokine expression related to TNFα production is a later but more prolonged phenomenon. It is likely that brain macrophages activated by T-cell-mediated pathway are the source of TNFα in TBM patients. This hypothesis is also supported by previous investigations that have demonstrated that *M. tuberculosis* cell wall components can trigger the release of TNFα from human and murine macrophages, as well as from pleural fluid mononuclear cells of patients with pulmonary tuberculosis.[5,15,16]

In conclusion, our data provide direct evidence that TNFα is produced and selectively concentrated for a long period of time in the CSF from patients with TBM. The chronic release of TNFα at the site of the infection suggests that this cytokine may be involved in the complex immunoregulatory mechanism that contributes to mycobacterial containment and elimination.

### References

1. Rosenblum MG, Donato NJ. Tumor necrosis factor α: a multifaceted peptide hormone. *Crit Rev Immunol* 1989; **9**: 21–44.
2. Havell EA. Evidence that tumor necrosis factor has an important role in antibacterial resistance. *J Immunol* 1989; **143**: 2894–2899.
3. Bermuduz LEM, Young LS. Tumor necrosis factor, alone or in combination with IL-2, but not IFN-gamma, is associated with macrophage killing of *Mycobacterium avium* complex. *J Immunol* 1988; **140**: 3006.
4. Barnes PF, Fong S-E, Brennan PJ, Twoney PE, Mazumder A, Modlin RL. Local production of tumor necrosis factor and IFN-γ in tuberculous pleuritis. *J Immunol* 1990; **145**: 149–154.
5. Takashima T, Ueta C, Tsuyuguchi I, Kishimoto S. Production of tumor necrosis factor alpha by monocytes from patients with tuberculous pleuritis. *Infect Immun* 1990; **58**: 3286–3292.
6. Glimaker M, Kragsbjerg P, Forsgren M, Olcén P. Tumor necrosis factor-α (TNFα) in cerebrospinal fluid from patients with meningitis at different etiologies: high levels of TNFα indicate bacterial meningitis. *J Infect Dis* 1993; **167**: 882–889.
7. Giusti G. Adenosine deaminase. In: Bergmeyer HU, ed. *Methods in Enzymatic Analysis*. Wienhen: Verlag Chemie, 1974; 1092–1099.
8. Leveton C, Barnass B, Champion B, *et al.* T-cell mediated protection in mice against virulent *Mycobacterium tuberculosis*. *Infect Immun* 1989; **57**: 390–395.
9. Ellner JJ, Wallis RS. Immunologic aspects of mycobacterial infections. *Rev Infect Dis* 1989; **2** (Suppl 2): S455–S459.
10. Orme IM, Miller ES, Robert AD, *et al.* T-lymphocytes mediating protection of mice against cytolysis during the course of *Mycobacterium tuberculosis* infection. Evidence for different kinetics and recognition of a wide spectrum of protein antigens. *J Immunol* 1992; **148**: 189–196.
11. Malan C, Donald PR, Golden M, Taljaard JJF. Adenosine deaminase levels in cerebrospinal fluid in the diagnosis of tuberculous meningitis. *J Trop Med Hyg* 1984; **87**: 33–40.
12. Ribera E, Martinez-Vazquez JM, Ocana I, Segura R, Pascual C. Activity of adenosine deaminase in cerebrospinal fluid for the diagnosis and follow-up of tuberculous meningitis in adults. *J Infect Dis* 1987; **155**: 603–607.
13. Adachi N. Beta-2-microglobulin levels in the cerebrospinal fluid: their value as a disease marker. *Eur Neurol* 1991; **31**: 181–185.
14. Wallis RS, Vjecha M, Amir-Tahmasseb M, *et al.* Influence of tuberculosis on human immunodeficiency virus (HIV-1): enhanced cytokine expression and elevated $\beta_2$-microglobulin in HIV-1 associated tuberculosis. *J Infect Dis* 1993; **167**: 43–48.
15. Moreno C, Taverne J, Mehlert A, *et al.* Lipoarabinomannan from *Mycobacterium tuberculosis* induces the production of tumour necrosis factor from human and murine macrophages. *Clin Exp Immunol* 1989; **76**: 240–245.
16. Barnes PF, Chatterjee D, Abrams JS, *et al.* Cytokine production induced by *Mycobacterium tuberculosis* lipoarabinomannan. Relationship to chemical structure. *J Immunol* 1992; **149**: 541–547.

ACKNOWLEDGEMENTS. This work was supported in part by a grant from M.U.R.S.T. (40% 1991), Rome, Italy. We thank Dr Pietro Gallo (ISS - COA, Rome) for help in statistical analysis.

**Received 5 November 1993; accepted in revised form 7 December 1993**
